# Buckle up safely: a cluster randomised trial to evaluate the effectiveness of a pre-school based program to increase appropriate use of child restraints

**DOI:** 10.1186/1471-2458-11-16

**Published:** 2011-01-06

**Authors:** Rebecca Q Ivers, Lisa Keay, Julie Brown, Lynne E Bilston, Kate Hunter, Judy M Simpson, Mark Stevenson

**Affiliations:** 1 The George Institute for Global Health, The University of Sydney, Sydney, Australia; 2 Sydney School of Public Health, The University of Sydney, Sydney, Australia; 3 Neuroscience Research Australia and The University of New South Wales, Sydney, Australia; 4 Accident Research Centre, Monash University, Melbourne, Australia

## Abstract

**Background:**

Road traffic crashes for car occupants are a leading cause of death and serious injury in children from high and middle income countries globally. Correct use of appropriate child restraints can significantly reduce death and serious injury but there is a need for well powered trials to examine effectiveness of programs to increase optimal child restraint practices. The aim of this trial is to examine the effectiveness of a comprehensive intervention to increase the use of appropriate child restraints, and decrease incorrect use of child restraints in pre-school aged children traveling in cars.

**Methods and Design:**

A cluster randomised controlled trial will be conducted, involving 28 pre-school or childcare centres in low income areas of Sydney, Australia, over one calendar year. The intervention is an educational program involving an in-service for centre staff, distribution of educational materials to parents, a parent workshop demonstrating restraint use, subsidised restraints for parents in need, and vouchers for a free restraint checking service. Blinded assessors will observe restraint use at all centres at the end of the calendar year. Data will be analysed on an intention-to-treat basis; the primary analysis will compare the proportion of each of the two outcome measures (use of appropriate restraints, and incorrect use of restraints) at each centre between intervention and control groups. Detailed process evaluation will be conducted, including examination of implementation and utilisation of various elements of the program by both centres and families.

**Discussion:**

This assessor blinded cluster randomised trial is powered to provide credible evidence about the efficacy of an education and distribution program in a pre-school setting to increase appropriate use, and decrease incorrect use of child restraints.

**Trial registration:**

Australian New Zealand Clinical Trials Registry ACTRN12609000612213.

## Background

Road traffic crashes are a leading cause of death and serious injury in children in high income countries worldwide, accounting for 41% of all child injury deaths [1]. Road traffic crashes for car occupants are a leading cause of death and serious injury in Australian children. There were 461 passengers under 12 years killed in traffic crashes between 1996 and 2005 in Australia[2], and about 1000 children aged 0-14 are seriously injured each year as car occupants[3], with about a third of these aged 0-4 [4].

The effectiveness of child restraint systems in preventing injury for children involved in traffic crashes is well documented. Data from the United States showed a 28% reduction in risk of death for children aged 2-6 years using child restraints [5]. For toddlers, Zaloshnja recently reported that the adjusted odds of injury were 82% lower for toddlers in child seats than for toddlers restrained in an adult seatbelt[6]. Children aged 4-7 years using booster seats had a 59% reduction in risk of injury compared to those in adult seatbelts[7]. An in-depth crash investigation study found that for children less than 14 years of age, dedicated child restraints performed best in terms of safety, followed by lap-sash belts, then lap-only belts[8]. A recent case-control study of 152 children aged 2-8 years found no serious or fatal injuries among optimally restrained children, while 30% of sub-optimally restrained children sustained serious or fatal injuries[9]. There is therefore clear evidence that children are at substantially lower risk of serious injury and death in the event of a car crash if they are restrained in an appropriate child restraint rather than an adult seatbelt.

However, for child restraints to be effective they need to be appropriate for the age (or height and weight) of the child. Inappropriate restraint use is defined as the use of a restraint by a child who is not suitable for that restraint on the basis of their age and/or size. It is recommended that children graduate to an adult seatbelt without a booster seat when a good fit is achieved, usually once children are 145-148 cm tall (about 11 years old)[10,11]. However, many children move to an adult seatbelt too early. A recent New Zealand study found that only 40% of children requiring a booster seat were using one, and in the 5-8-years age group, 93% required a booster seat but only 30% were using one [12]. Other studies have shown that inappropriate restraint is highest among children aged 2-8 years [13-15]. Recent Australian research showed that, of children meeting the booster seat height-weight criteria, 56% had been prematurely graduated into an adult seat-belt[16].

Factors that have been associated with premature graduation from one restraint type to another in recent Australian studies include low parental education [16-18], poor parental knowledge of appropriate restraint transition points [18], larger families [16,18], older age of child [18], travelling in other people's vehicles and parenting style [18]. Studies conducted in the US have identified perceived restraint comfort, lower income status and educational attainment as factors associated with a higher risk of early graduation to a seatbelt-only restraint [19,20].

Although children may be restrained in an age-appropriate child restraint, incorrect use of restraints can also degrade the level of protection provided in a crash. Incorrect use is when the restraint system is not used as intended and can occur in both child restraints and adult seatbelts with or without booster seats. Examples include incorrect positioning and/or tension of the belt and incorrect attachment of the restraint to the vehicle. Incorrect restraint use substantially increases injury risk. In its most serious form, an incorrectly used restraint may fail to control the child's motion in a crash, leading to impact with the vehicle interior and other occupants. An Australian retrospective case review of 2-8 year olds in crashes found that children incorrectly using restraints were 6.9 times more likely to be seriously injured than those correctly using their restraints, irrespective of restraint type [9]. More recent work has shown that 51% of children in the state of New South Wales (NSW) are incorrectly restrained, with the prevalence of incorrect use highest among those using dedicated child restraints (65% of those using forward facing restraints and 60% of those using booster seats, compared to 48% of those using adult seatbelts). In addition, the data have shown that the forms of incorrect use were more serious for children restrained in seatbelt, booster seats and harnesses, all typically used by children in the 3-6 year age group [21]. Risk factors for incorrect use of child restraints include lower educational levels, families who remove the seat frequently from their vehicle, drivers who are not the parent of the child, and young, small children [22]. Incorrect use of booster seats is more likely to occur in children from low socioeconomic and non-English speaking background (NESB) families [23].

Previous research has predicted that the greatest gains are to be made by targeting both the use of appropriate restraints and the correct use of restraints [24]. Studies have also demonstrated the effectiveness of various interventions to increase child restraint use. A recent systematic review [25] found that incentives or the provision of a free child restraint combined with education increased the use of restraints - but concluded that, although the interventions seemed promising, there was a need for high quality well-controlled studies to evaluate effectiveness of programs aimed at increasing use of child restraints. A recent US trial found that a pre-school based intervention to increase restraint use was not effective but also reported that few families received the intervention as planned [26]. Intervention studies aimed at decreasing incorrect use of restraints are less common, with only one published study, which showed an education program was effective in increasing correct use of restraints [27]. There have been no Australian trials examining effectiveness of interventions to increase the use of appropriate child restraints or decrease incorrect use of child restraints.

There is a clear need for a well powered trial of interventions aimed at improving the quality of restraint use. Given the lack of effectiveness of previous trials, there is also a need to understand clearly the process aspects of the trial.

The aim of this trial is therefore to examine the effectiveness of a comprehensive intervention to 1) increase the use of appropriate child restraints and 2) decrease incorrect use of child restraints in pre-school aged children travelling in cars.

## Methods

The study is a cluster randomised trial, designed in line with the CONSORT statement [28], with randomisation of 28 pre-school or childcare centres. Eligible centres will be identified from early childhood education services in the Sydney metropolitan area via the Kids and Traffic program at Macquarie University who provide road safety education services to all pre-schools and childcare centres state-wide. Directors of eligible centres will be approached by mail and phone by research staff to participate in the trial. Once the directors have consented to join the study and to be randomised, randomisation will occur into treatment and control groups.

Eligible centres will include community pre-schools or childcare centres with a minimum of 20 families with a child in the eligible age range (3-5 years). Children with their 3^rd ^birthday in 2010 and older will be included as inappropriate use begins to increase dramatically from this age and 3 year old children are widely enrolled in pre-school programs. Because interventions are most needed in families from low educational backgrounds, eligible centres will be those based in local government areas (LGA) of Sydney metropolitan area ranked in the lowest 30% of LGAs in the Sydney Metropolitan Area according to the Socioeconomic Indices For Areas (SEIFA): Index of Education and Occupation scores (SEIFA scores <980). LGAs to be included are Fairfield, Campbelltown, Liverpool, Blacktown, Canterbury, and Bankstown. English must be used as the instructional language in the centre and physical layout of the centre and parking must allow for safe observation of vehicles upon arrival, without major disruption to traffic flow. Finally, centres must have no active engagement or specific policies on child restraints, or programs where they actively engage with families on this issue. A complete list of eligible centres will be created, stratified by service type (pre-school or long daycare), and each will be allocated a unique identifier. The centres will be approached in random order until enough centres of each service type have been recruited. The number of each service type will be chosen to match the distribution of service types in these areas, with an even number of each type and totalling 28. An authorised representative of the centre will sign a record of informed consent.

A baseline assessment will be conducted before randomisation to determine whether the control and intervention groups have similar baseline restraint use patterns. While direct observation of children in situ would be the best way to measure baseline levels of appropriate restraint use, such observation and necessary contact with research assistants may in itself act as an intervention. To avoid such potential contamination of the control group, baseline measures will therefore involve a brief self-report questionnaire to be completed by parents/guardians in both intervention and control centres when they sign children in or out of the centre. Information will be collected about number of children in the family, age of children, type and frequency of restraint use and seating position in car. Although this technique will be less accurate than the direct observations made post intervention, previous work has shown that parent interview tools may be used to accurately describe child restraint use [29], and it has been determined that the risk of measurement error is less important than any possible contamination of the control group which may occur by more detailed measures. This process will also remove any ethical dilemmas that may arise if children in the control group were observed to be restrained inappropriately. Any self-report bias should be similar between groups.

Restricted randomisation will be conducted using the unique identifiers and summary data from the baseline survey to ensure adequate balance of important factors. The five balance criteria will be (i) service type exactly balanced between groups; (ii) self-reported restraint choice for the 3-5 year-old children attending the service (proportion appropriately restrained): mean proportions in each group differ by no more than 5% in relative terms (i.e. ratio of means between 0.95 and 1.05); (iii) mean proportion of families with annual household income below AU$60,000 in each group differ by no more than 10% relatively; (iv) mean proportion of families who speak a language other than English at home in each group differ by no more than 10% relatively; and (v) mean size (number of children aged 3-5 years enrolled) of centres in each group differ by no more than 10% relatively. The randomisation process will be fully concealed as the statistician will allocate the centres at a site remote to the recruitment site and will only have access to a list of centres recruited. Of the 28 centres in the study, 14 centres will be randomly allocated to each of the intervention and control groups.

### Intervention

The intervention will be a comprehensive education and restraint distribution program, to be delivered in pre-school settings under the Kids and Traffic program. The Kids and Traffic program offers a road safety orientated education service to all child care services in NSW. Funded by the Roads and Traffic Authority (RTA) of NSW, the 3200 licensed early childhood services registered with the program are sent road safety information, programming advice, posters and information to share with families at least once each year. It has developed a range of strategies, based on the principles of early childhood learning and best practice in early childhood education which support road safety education in pre-school/daycare settings. These include professional development workshops primarily for staff and others designed for both staff and families. The program will be enhanced as part of this trial such that specific material will be developed around increasing use of appropriate child restraints and decreasing incorrect use of child restraints. In line with interventions tested in smaller trials in other settings [25,30], this material will include an educational component (including staff and parent workshops), and will be further enhanced by distribution of child restraints at reduced cost and ongoing distribution and reinforcement of educational materials at the centre level.

The program to be tested in the trial includes the following components:

1. Centres will receive the enhanced workshop from the Kids and Traffic program, with the focus on restraint use. This will require in-service training for staff, and resources for incorporating messages about appropriate restraint use into programming for the centre. Centre staff will also receive monthly policy support from the Kids and Traffic Program staff, and will be provided with extra printed information that can be incorporated into parent newsletters to reinforce messages.

2. Educational material will be provided to intervention centres, consisting of pamphlets and posters about optimal child restraint practices, and information about frequently asked questions, with photographs of appropriate use and incorrect use. Written material will be translated into a range of community languages. These materials will contain specific information about what types of restraints should be used by children in the target age group, and common forms of incorrect use and the impact these have on crash safety. Centres will be asked to display restraint information provided in prominent positions. Approximately 6 weeks following the staff inservice, a parent information session will be held at each centre. This session will include a presentation by the Kids and Traffic personnel, and will include viewing of a DVD created for the trial detailing the benefits of appropriate and correct restraint use and reinforcing recommendations for correct and appropriate use provided in print material, as well as hands-on demonstrations of child restraints. All parents enrolled at each intervention centre will receive material on restraints including the DVD, brochures on appropriate restraints, and written and pictorial information on restraint use. All families will be given a free voucher for a visit to an authorised child restraint fitting station for personalised advice on correct restraint use and will receive information on location of fitting stations in their area.

3. Subsidised child restraints will be made available at a minimal cost ($AUD50, approximately 25% of recommended retail price) for those who require them.

The control group will be offered the standard Kids and Traffic program rather than the enhanced program described above. The current program offers minimal information about use of child restraints so, even if implemented, it is unlikely to have a significant impact on appropriate and correct restraint use. Control sites will be given the option of receiving the enhanced Kids in Traffic workshop at the end of the trial.

Outcome measures will be both use of appropriate restraints for the age of the child, and incorrect use of restraints, and will be collected 2 months after the intervention has been implemented using a proven observational method [21]. Masked observers will attend each site over a 1-2 hour period during morning drop-off times. Potential participants will be approached as they pull into a parking spot outside the institution, and informed consent will be gained from all participating parents. Initial observations will be made with the child in situ. The driver of the vehicle will then be invited to participate in a brief interview. All refusals will be recorded and brief observational and demographic data collected to allow evaluation of potential bias between participants and non-participants. A brief structured interview will be conducted with those consenting to record parent/guardian age, gender, education, and information about age and gender of child, usual child restraint use, number and age of children in family. A detailed examination of the restraint installation by trained observers will be conducted while the interview is taking place.

Although it would be ideal to measure outcome after a longer time period, it is difficult in practice to follow-up children over two calendar years. Most of the children aged 4-5 at the beginning of the intervention year will move to primary (elementary) school the following year. It would not be practical to collect follow-up data from children at this time as they will move to a range of different schools and direct observation will not be feasible. While 6 month follow-up data could be collected by phone, likely differential reporting bias would mean that such outcome data would be severely compromised.

In order to assess compliance and uptake of the program, records will be kept of which families attend the education sessions, and receive individualised fitting services or subsidised child restraints. Information about alternative road safety or restraint programs administered by either intervention or the control sites will be collected. Importantly, as understanding practical issues about how to implement such programs is vital to further roll-out of successful programs, process measures will also be evaluated by asking the director and staff of the intervention group centres to complete a short questionnaire about the implementation of the program.

Research assistants conducting the pre-and post-trial observations will be blinded to the intervention/control status of each centre. Furthermore, research staff will be trained appropriately and outcome measurements will be based on standardised objective criteria, minimizing opportunities for measurement error. As pre-trial observations are collected by self-administered questionnaires, research staff will not observe children until after the intervention has been implemented. For both intervention and control centres, research staff conducting post-trial observations will present parents or guardians of children with written information about appropriate use of child restraints and offer advice if any child is observed in inappropriate restraints or using restraints incorrectly. Appropriate bilingual staff will be employed to conduct baseline interviews, implement the program and conduct post-trial observations, to meet the language needs of participating families.

On 1 March 2010 new national child restraint laws were enacted in NSW, mandating appropriate restraint use by children up to age 7. Enforcement of these laws was delayed until 30 June 2010 to allow parents and carers time to fully understand and comply with the new laws. The new laws will impact on all pre-school centres and parents in the trial. The impact of the new laws is that the baseline rate of appropriate child restraint use may increase in all centres, whether control or intervention. It is unlikely that the rate of incorrect use will change as the social marketing and education campaigns behind the new laws are focused more on use of appropriate restraints rather than on reducing incorrect use. Baseline interviews will be conducted before the enforcement of the new law at all centres, and for most centres before the 1 March introduction.

### Sample size and statistical analysis

This study has 80% power to detect a 20% increase in use of appropriate child restraints from 60% to 80%, as significant at the 5% level. Previous research has indicated that educational and incentive based interventions may increase use of restraints by about 20% [25]. In NSW the use of appropriate child restraints in 20 centres in Sydney varied from 30-70% [31]. However, among centres from local government areas with low educational attainment, the average use of appropriate child restraint use is 30-40%. If the effect of the implemented RTA general marketing program raises the base rate in controls to 60%, the study will be adequately powered to find an effect of a 20% increase. However, it will also have power to detect a 20% effect if the base rate of restraint use remains between 30-50%. Increasing the rate to 80% is feasible: the base rate of child restraint use in centres in areas with high education and high socioeconomic status is around 70%, so this increase is possible. Previous work has allowed estimation of the intra-class correlation as 0.06, based on proportion using appropriate restraint at 9 centres [31]. Allowing for a minimum of 20 eligible children at each centre (with an average number of slightly more than 20 anticipated) would require 20 centres. Further allowing for 30% centre drop-outs through the course of the study requires 14 intervention centres and 14 control sites. This sample size will also allow 80% power to establish whether incorrect use of restraint systems can be reduced from 60% of children using restraints to 40%. Direct observation of children in pre-school settings in Sydney found that 65% of children using forward facing child restraints and 60% of children in booster seats were incorrectly using the restraints [31].

Data will be analysed on an intention-to-treat basis. The primary analysis will compare the proportion of each of the two outcome measures (appropriateness and correctness of restraint use) at each centre between the intervention and control groups using Donner's adjusted chi-square test[32]. Logistic regression modelling will be used to explore predictors of each outcome measure, using generalised estimating equations (GEE) to allow for clustering of data by centre.

The study protocol was approved by the Human Research Ethics Committees of the University of Sydney and the University of NSW.

## Discussion

This assessor blinded cluster randomised trial is powered to provide credible evidence about the efficacy of an education and distribution program in a pre-school setting to increase appropriate use, and decrease incorrect use of child restraints. The intervention is an evidence based program that could be implemented on a larger scale if found to be effective, via the existing, government funded, Kids and Traffic program. The study results will also highlight whether such programs can increase appropriate restraint use over and above the increases found as part of new enforcement, education and legislation introduced simultaneously, and further, whether it is possible to offset the higher rates of incorrect use associated with increased use of appropriate restraints. The results of this study will therefore have major relevance to policy makers and child safety advocates as well as researchers in both Australia and internationally. If such interventions can be shown to be efficacious, they show great promise for implementation. The results will also be of great relevance to researchers and policy makers in the US and other high income countries.

## Competing interests

The authors declare that they have no competing interests.

## Authors' contributions

RI, LB, JB and LK led the methodological design of the study, supported by JS, KH and MS. RI and LK drafted the paper and all authors contributed to revisions. All authors read and approved the final manuscript.

## Pre-publication history

The pre-publication history for this paper can be accessed here:

http://www.biomedcentral.com/1471-2458/11/16/prepub
